# Clinic Atlanta Medical College

**Published:** 1873-12

**Authors:** A. W. Calhoun

**Affiliations:** Professor of Diseases of the Eye and Ear


A. W. CALHOUN, M.D.,
*Professor of Diseases of the Eye and Ear.*

Case 1.—A. R., colored, 49 years of age, from Morgan county, Georgia, presented himself for treatment, on November 5th, 1873, with cataract, of hard variety, in each eye. The left eye was at once operated upon; Graefe's linear extraction, with conjunctival flap being the method adopted. The eye was then dressed, a plain, ordinary bandage being applied, and this was changed twice daily during treatment of the case.

November 12th. Right eye operated upon in same manner.

Left eye progressed nicely to recovery, nothing unusual occurring during healing of the parts. Small portion of cortical substance of lens was left behind in the second operation, but this was almost entirely absorbed before the patient's dismissal. He was discharged on Nov. 25th with excellent vision, using double convex glasses, No. 3½. for each eye. Owing to his inability to read he was not tested with test types, but with other test objects. Vision ⅔.

Case 2.—Miss A. B., white, twenty-two years of age, chambermaid. November 22d, came to clinic with an opacity of cornea of the right eye, the result of an inflammation which had totally destroyed her sight in that eye. Treated by tattooing, using India ink and tattooing needles in the operation. Considerable inflammation followed the treatment, which was quite unexpected, being unusual, as in nearly all cases no irritation whatever attends such an operation. The deformity was entirely removed. No attempt was made to restore vision, as this had been destroyed by the original inflammation.

Case 3.—November 22d, Oliver B., æt. fifty-two years, negro, married, a well-digger. Diagnosis: Gonorrhœal opthalmia. Lower lid of left eye everted and conjunctiva exposed, forming a semi-solid tumor. Had gonorrhœa just previous to this trouble, and penis is still discharging a little, though he denied being affected with the disease. Eye-lids much swollen, both eyes being implicated. Cornea of right eye has sloughed off entire, causing a jutting forward of iris, which is probably the beginning of staphyloma. Ulceration also of left cornea, but portion still remaining intact, and perforations having commenced to heal, give some hope of saving sight in that eye. Treatment:

℞.—Argenti Nitratis, . . . . . . gr. x.

Aquæ Puræ, . . . . . . f℥j.

Misce, and paint lids once, twice or thrice daily, according as the white eschar sloughs off; the application to be made with a camel's hair pencil. General attention to cleanliness was also enjoined upon the patient, and he was cautioned to burn all cloths or sponges used in bathing the eyes, to prevent communicating the disease to others. Up to December 13th he was rapidly and steadily improving under this treatment. It is the intention, when the inflammation subsides, to make an iridectomy on left eye, with a view of rendering that eye sufficiently useful to enable him to get about.

Case 4.—November 26th, Jack R., æt. forty-eight years, colored, a wood-sawyer. This man is suffering from too long continued and too free use of nitrate of silver on conjunctivæ for the cure of some conjunctival affection. Has used the nitrate in very strong solution for many months, producing Argyrosis. Conjunctival membrane is of a dirty, dish-water hue, and the pupil of one eye is much distorted by anterior synechia. Opthalmoscopic examination revealed complete atrophy of optic nerve in both eyes. Nothing attempted for disease of the optic nerves, as the pathological condition is beyond remedy.

Case 5.—November 29th. Henry W., aged six years, negro. Opthalmia Pustulosa affecting the right eye. Treated by dropping into the lower conjunctival fold a solution of sulphate of atropia—one grain to the ounce of water, and using two or three drops at a time until dilatation of the pupil is complete, and then continuing the use of the same remedy twice daily. Also,

| | |
|---|---|
| ℞.—Hydrarg. Oxidi Flav. . . . . | grs. iss. |
| Cerati Simplicis, . . . . . . | ℥ j. |

Mix and apply a small amount, the size of a pea, once daily to conjunctiva of inferior palpebrum. December 3d, case progressing favorably. Continue treatment until cured, and for two or three weeks thereafter to prevent a relapse, December 13th—has been doing well up to date. On the 10th instant was nearly well, but discontinued the application of remedies contrary to instructions, and to-day has the disease again in all its former severity, with ciliary neuralgia, photophobia and ciliary injection. Former course of treatment re-commenced and ordered to be strictly persevered in.

Case 6.—November 29, C.W. H., white, farmer, from Barbour county, Alabama, forty-three years of age. Cataract; mature; in the right eye. Sight of left eye entirely destroyed by an injury several years ago. Cataract extracted as in case No. 1. Vision tested by fingers in usual way immediately after the extraction. Discharged on the 13th of December, entirely cured; no troublesome symptoms having followed the operation. When tested, at that time vision was found to be $\frac{20}{30}$ with convex glass 4½, and he could read Snellen's test types 1½ at 8 inches, with convex glass 2½.

Case No. 7.—Mr. T. J. C., a member of the medical class, æt. twenty-four years, Butts county, Ga. A pterygium exists in both eyes, growing from plica semi-lunaris, and extending by small point over edge of cornea. These were treated, the one by cutting out a wedge-shaped piece of the membrane, and approximating edges of denuded surfaces by a couple of silk sutures; the other by strangulating at the base, by means of ligatures, under surface and apex of the pterygium.

Case No. 8.—Mrs. L. C., white, thirty years old, washerwoman, mother of several children. Diagnosis: retinal hemorrhage. Came before class on December 8th. Has been troubled with her eyes since the birth of her last child. At that time had very severe ciliary neurosis, especially in frontal region, and dizziness; also was disturbed with mouches volantes. External appearance of eye is normal. Upon ophthalmoscopic examination, however, seious internal derangement is discovered. Numerous extravasations of blood exist all over surface of retina—one very large, partially covering the optic disk. Ordered to take calomel in small doses, combined with Dover's powder, every few nights. For frontal neuralgia, the following is prescribed:

| | | |
|---|---|---|
| ℞.—Pulvis Opii, | . . . . . | gr. iv. |
| Unguenti Hydrarg., | . . . | ℨij. M. |

Apply to surface over painful part by brisk rubbing ter in die.

December 13th—Further examination discovers that the hemorrhagic spots are being absorbed, and have greatly diminished in size. Vision of patient is decidedly defective.

Case No. 9.—December 10th—Mr. O. T. D., medical student. Inflammation and obstruction of lachrymal sac existed in this case, the left side being the one diseased. It was treated by the Professor, who slit up the lower canaliculus of the corresponding eye with a Weber's knife, and then introduced a Bowman's probe (No. 1) through the nasal duct. Operation of passing probe must be performed daily for some weeks, to effect a permanent cure of the stricture.

Case No. 10.—Lula W., colored, seven years of age. Was brought to clinic on the 10th of December, suffering from conjunctivitis pustulosa, with excessive photophobia. The disease is dependent upon a scrofulous taint of patient's system. To relieve the intolerance of light, and assist in reducing the inflammation, Dr. Calhoun ordered her put upon the following prescription:

| | | |
|---|---|---|
| ℞.—Hydrarg. Precipitat. albi, | . . | gr. vi. |
| Extracti Belladonnæ, | . . . | gr. viij. |
| Cerati Simplicis, | . . . . | ℥j. |

M. et ft. unguentum. To be applied by thoroughly rubbing over forehead every three hours during the day.

In addition to the foregoing, a solution of sulphate of atropia—grs. j to f℥j of water—was used by dropping into the eye every two or three hours, and patient was enjoined to partake of very nourishing food, to improve general condition of system.

Case No. 11.—December 10th—Jacob M., white, æt. thirty-eight years; merchant by profession. This man has a recent and serious disease of the left eye—acute glaucoma. It has existed but a few days and has progressed rapidly to loss of vision in the affected eye, but by a simple operation the progress of the disease can be arrested. The case was promptly taken in hand, and the Professor treated it by making an iridectomy at once. After treatment consists simply of ordinary bandage and attention to cleanliness of the eye.

